# Identifying trigger cues for hospital blood transfusions based on ensemble of machine learning methods

**DOI:** 10.1186/s12245-024-00650-0

**Published:** 2024-06-19

**Authors:** Eva V. Zadorozny, Tyler Weigel, Samuel M. Galvagno, Christian Martin-Gill, Joshua B. Brown, Francis X. Guyette

**Affiliations:** 1 University of Pittsburgh, Graduate School of Public Health, 4420 Bayard Street, Suite 616-12, Pittsburgh, PA 15213 USA; 2 University of Pittsburgh School of Medicine, Pittsburgh, PA USA; 3 Department of Anesthesiology, University of Maryland Medical Center, R Adams Cowley Shock Trauma Center, Baltimore, MD USA; 4 Department of Emergency Medicine, University of Pittsburgh School of Medicine, Pittsburgh, PA USA; 5 Department of General Surgery, University of Pittsburgh School of Medicine, Pittsburgh, PA USA

**Keywords:** Prehospital transfusion, Early hospital transfusion, Hemorrhagic shock, Prehospital lactate concentration, Fast frugal trees, Bayesian analysis, Decision support models

## Abstract

**Background:**

Traumatic shock is the leading cause of preventable death with most patients dying within the first six hours from arriving to the hospital. This underscores the importance of prehospital interventions, and growing evidence suggests prehospital transfusion improves survival. Optimizing transfusion triggers in the prehospital setting is key to improving outcomes for patients in hemorrhagic shock. Our objective was to identify factors associated with early in-hospital transfusion requirements available to prehospital clinicians in the field to develop a simple algorithm for prehospital transfusion, particularly for patients with occult shock.

**Methods:**

We included trauma patients transported by a single critical care transport service to a level I trauma center between 2012 and 2019. We used logistic regression, Fast and Frugal Trees (FFTs), and Bayesian analysis to identify factors associated with early in-hospital blood transfusion as a potential trigger for prehospital transfusion.

**Results:**

We included 2,157 patients transported from the scene or emergency department (ED) of whom 207 (9.60%) required blood transfusion within four hours of admission. The mean age was 47 (IQR = 28 – 62) and 1,480 (68.6%) patients were male. From 13 clinically relevant factors for early hospital transfusions, four were incorporated into the FFT in following order: 1) SBP, 2) prehospital lactate concentration, 3) Shock Index, 4) AIS of chest (sensitivity = 0.81, specificity = 0.71). The chosen thresholds were similar to conventional ones. Using conventional thresholds resulted in lower model sensitivity. Consistently, prehospital lactate was among most decisive factors of hospital transfusions identified by Bayesian analysis (OR = 2.31; 95% CI 1.55 – 3.37).

**Conclusions:**

Using an ensemble of frequentist statistics, Bayesian analysis and machine learning, we developed a simple, clinically relevant prehospital algorithm to help identify patients requiring transfusion within 4 h of hospital arrival.

**Supplementary Information:**

The online version contains supplementary material available at 10.1186/s12245-024-00650-0.

## Introduction

Hemorrhagic shock is the leading cause of preventable death among injured patients [1]. Shock occurs in a continuum with progressive end-organ damage and leads to death if inadequately treated. Aggressive resuscitation according to damage control principles reduces the risk of death from hemorrhagic shock [2]. Damage control resuscitation with prehospital blood products lowers the risk of death, although the role for prehospital blood remains unclear [3, 4]. Early resuscitation prevents the consequences of hemorrhagic shock and poor outcomes but is difficult to achieve in the prehospital environment with constrained diagnostic and therapeutic capabilities. Current field triage guidelines use vital signs and level of consciousness to determine the need for expedient transport to a trauma center, but these guidelines may overlook many patients with unrecognized or compensated shock who may benefit from early blood administration [5]. Indications for prehospital blood transfusion after injury vary considerably and rely on arbitrary vital sign thresholds and obvious symptoms of hemorrhagic shock [6].

Prior work shows that elevated serum lactate levels in trauma patients may indicate sepsis and multiorgan dysfunction, increasing the chance of mortality [7, 8]. Prehospital clinicians can measure serum lactate levels using rapid, relatively inexpensive point of care tests to guide current triage decisions in the case of serious injury. In our previous work, we found that increased prehospital lactate levels were associated with higher odds of 24-h hospital transfusion, even among patients without hypotension [5]. Prehospital lactate may be a useful prompt for prehospital transfusion. To mitigate significant physiologic derangement, prehospital professionals need a reliable but simple approach to rapidly and accurately identify patients who are most likely to benefit from prehospital blood. Our objective was to develop a parsimonious clinically relevant algorithm to identify patients requiring early hospital transfusion using data available in the prehospital setting. This algorithm may be a guide for prehospital blood product administration.

We hypothesized that using state of the art statistical techniques to control for known confounders, we would identify a subset of factors highly predictive of transfusion need after injury, thereby creating a simple in-field operational model for identifying patients who need blood during trauma resuscitation. We aimed to compare the accuracy of data-driven methods with conventional triage criteria thresholds to determine variables with the optimal sensitivity and specificity for identifying trauma patients who require a blood transfusion. We also aimed to develop proof of concept decision models with components that could be adapted to different prehospital services such as rural versus urban settings.

## Methods

We performed a retrospective analysis of prehospital factors that predict the need for emergent blood administration (within 4 h) in adult (age > 16 years) trauma patients. The hours were calculated as number of minutes between ED arrival and discharge dates divided by 60. These dates are electronic timestamps. We included trauma patients with recorded venous lactate who were transported by a regional critical care transport service between 2012 and 2019. We excluded subjects with isolated traumatic brain injury (TBI) (18.6%), those that died in the emergency department (0.4%), and those with missing data (< 4%). Isolated TBI was defined as head abbreviated injury scale (AIS) and no other severe injuries (AIS face, neck, chest, spine, arms, abdomen, legs, external < = 2) as these patients are not likely to require transfusion. The University Human Research Protections Office approved this study.

The data was from a regional critical care transport service that has 18 helicopter and 2 ground bases across four states. Blood is available at all bases; 2 units of PRBCs is taken by helicopters on all missions. Crews complete 13,000 missions annually and include a minimum of a critical care nurse and paramedic. They are trained to perform point of care testing for blood gases and lactate concentration (iSTAT One, CG4 + , Abbott Laboratories Princeton, NJ). They use these data to inform resuscitation and titrate mechanical ventilation.

To build an operational in-field model to identify the need for blood use, we used an ensemble of methodologic approaches. Our first approach was to construct Fast and Frugal Trees (FFTs) using prehospital factors associated with hospital blood administration, previously identified using logistic regression as influencing hospital blood decisions (Table 1) [5]. Factors associated with hospital blood administration were used to find data-driven thresholds. The algorithm that builds FFTs compares FFT receiver operating characteristics to those of other common model-building approaches: CART, logistic regression, Random Forest (RF) and Support Vector Machine (SVM) methods (see Appendix) [9].

**Table 1:** Cohort characteristics

Variable	All Subjects (*n* = 2,157)	4 h hospital ED blood products		
		Yes (n = 207; 10%)	No (n = 1,950; 90%)	P value^**♦**^
Prehospital venous lactate (mmol/L)	2.71 (1.40 – 3.15)	4.85 (2.30 – 5.80)	2.48 (1.30 – 2.98)	< 0.01
Age (years)	47 (28 – 62)	49 (28– 65)	47 (29 – 62)	0.26
Sex (male)	1,480 (69)	141 (68.0)	1,339 (68.7)	0.88
CCI	0.67 (0 – 1)	0.78 (0 – 1)	0.66 (0 – 1)	0.62
ISS	11 (4–14)	20 (10 – 29)	9.55 (4 – 13)	< 0.01
Lowest SI	0.59 (0.47 – 0.68)	0.74 (0.56 – 0.89)	0.58 (0.47 – 0.67)	< 0.01
SI range	0 (-0.08 – 0.08)	-0.02 (-0.15 – 0.14)	0 (-0.07 – 0.07)	0.68
Lowest SBP	115 (100 – 132)	85 (67 – 102)	118 (103 – 133)	< 0.01
SBP < 90 mmHg^a^	345 (16)	129 (62)	216 (11)	< 0.01
Heart rate > 120 bpm^a^	442 (20)	79 (38)	363 (19)	< 0.01
Blood prior to EMS (ml)	37 (0 – 0)	110 (0 – 0)	29 (0–0)	< 0.01
Blood by EMS (ml)	27 (0 – 0)	173 (0 – 300)	12 (0 – 0)	< 0.01
Crystalloids prior to EMS (ml)	418 (0 – 500)	793 (100 – 1,000)	378 (0 – 500)	< 0.01
Crystalloids by EMS (ml)	201 (50 – 200)	173 (0 – 300)	165 (50 – 150)	< 0.01
Transfer^ac^	931 (43)	90 (43)	841 (43)	0.94
Penetrating^ad^	206 (10)	31 (14)	175 (9)	< 0.01
AIS head > 2^a^	199 (9)	51 (25)	148 (8)	< 0.01
AIS chest > 2^a^	550 (26)	95 (46)	455 (23)	< 0.01
AIS spine > 2^a^	148 (7)	15 (7)	133 (7)	0.77
AIS abdomen > 2^a^	28 (1)	4 (2)	24 (1)	0.34
AIS legs > 2^a^	311 (14)	62 (30)	249 (13)	< 0.01
Volume of hospital blood (ml)^b^	497 (0–0)	3,610 (600 – 4,600)	167 (0 – 0)	< 0.01
Mortality^ab^	34 (2)	19 (10)	15 (1)	< 0.01
Laparotomy^ab^	319 (15)	125 (60)	194 (10)	< 0.01
Thoracotomy^ab^	274 (13)	81 (39)	193 (10)	< 0.01
Craniotomy^ab^	34 (2)	13 (7)	21 (1)	< 0.01
Interventional radiology^ab^	125 (6)	26 (13)	99 (5)	< 0.01
Pelvic fixation^ab^	9 (0.4)	4 (2)	5 (0.3)	< 0.01
Vascular repair^ab^	38 (2)	12 (6)	26 (1)	< 0.01

^a^n (%) shown for categorical variables, median (IQR) shown for continuous variables

^b^Within 24 h of hospital admission

^c^ The rest of population was transported from scene

^d^The rest of population had blunt injuries

^♦^Means for categorical variables were compared using Fisher’s exact test, for continuous variables – using Mann–Whitney U test

We implemented FFT and Bayesian approaches as independent yet complimentary methods that validate each other’s findings. A heuristic (rule of thumb) FFT approach minimizes variance but is more prone to bias [9], whereas a Bayesian approach is less biased and more prone to higher variance [10]. Using both FFT and Bayesian approaches minimize the overall error from both bias and variance.

FFTs are decision trees that differ from conventional decision trees in three ways: 1) they contain a minimal number of variables/cues needed to decide, 2) they make a decision after every node, and 3) they can only have two branches per node [9]. These trees are salient (we know how the machine arrived at the decision), robust against overfitting and good at identifying new cases of the outcome variable. This makes FFTs ideal to guide fast decisions in dynamic and dangerous environments [9]. We split the analysis data set 50/50 into training and testing datasets (a common starting point for evaluating machine learning algorithms) [11] and applied the FTT algorithm. For more information about the FFT algorithm, please see the Appendix/Supplemental Methods section.

Our second approach was a Bayesian analysis of factors predicting in-hospital transfusion to confirm or supplement our prior approaches. Our goal was to identify a parsimonious model to predict transfusion within 4 h of hospital admission. A Bayesian approach was employed for several reasons. First, prior information from our group and others may be used to provide updated knowledge about variables most strongly associated with the probability that a trauma patient requires a blood transfusion. Second, a hazard with frequentist statistics is that P values and confidence intervals may be difficult to interpret; highly significant P values may not be clinically meaningful or intuitively comprehensible. Third, Bayesian methods yield the probability of a specific outcome given the data [10].

Finally, we synthesized the results of our approaches to create a proposed clinical algorithm of indications for prehospital blood transfusion.

This work adheres to STROBE guidelines of reporting in observational studies (Appendix Table 1). Data analysis was performed using R® version 4.1.2 (Vienna, Austria), SAS® version 9.4 (Carry, NC), and Stata® version 17 (College Station, TX).

## Results

**Fig. 1:**
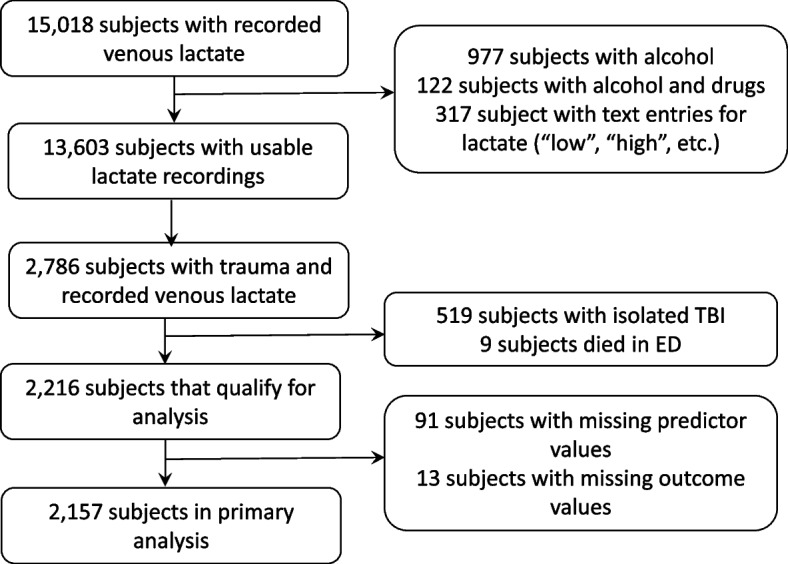
Flow diagram illustrating cohort selection. STROBE guidelines are shown in grey rectangles. Subjects with trauma were received in a trauma or burn unit and/or had the following mechanisms of injury: assault, animal bite, burn, electrocution (non-lightning), gunshot wound, stabbing/cutting, machinery accident; pedestrian, bicycle, motor vehicle, all-terrain vehicle, motorcycle, water transport, or aircraft accident, crash or collision

Of the patients transported over the seven-year study period, we identified 2,157 trauma patients with a prehospital lactate value (Fig. 1) obtained according to the Blood Administration protocol (Supplemental Table 1 and Appendix 2).

Among the cohort, 1,480 (68.6%) patients were male, mean age was 47 (IQR = 28 – 62), and 207 (9.60%) patients had the primary outcome of requiring a blood transfusion within 4 h of admission to the Emergency Department (Table 1).

The median prehospital lactate concentration was 4.85 mmol/L for the subjects who received blood products (IQR = 2.30–5.80), and 2.48 mmol/L for the subjects who did not require hospital blood products within 4 h of arrival (IQR = 1.30 – 2.98). Of the subjects who received hospital blood products, 19 (10%) died within 24 h of admission. Only 1% of the subjects who did not require hospital blood died within 24 h of admission (*n *= 15). Consistently, a greater percentage of subjects who received hospital blood products needed other hospital life-saving interventions (LSIs) (Table 1).

We excluded information about prehospital blood and crystalloids given by the prehospital care service and prior to arrival from the decision process because of significant collinearity (i.e., relationship between model predictors) related to in-hospital blood administration. We provided the FFT algorithm with 13 variables to choose from based on clinical value and availability to the prehospital clinicians [12]. Among them were AIS scores provided as a surrogate for injury condition that is visible to prehospital clinician, which we also previously found to associate with hospital transfusion. While we acknowledge the AIS value would not be available in the prehospital setting, we use them here as a proxy for clinically recognizable anatomic injury patterns that are used in the field by EMS clinicians for trauma triage purposes. Five of the thirteen variables were not selected by the algorithm as they were not associated with need for blood transfusion: 1) critical high heart > 120 bpm, 2) AIS abdomen > 2, 3) AIS spine > 2, 4) injury type (blunt or penetrating), and 5) shock index (SI) range (i.e., difference between highest and lowest SI).

**Fig. 2:**
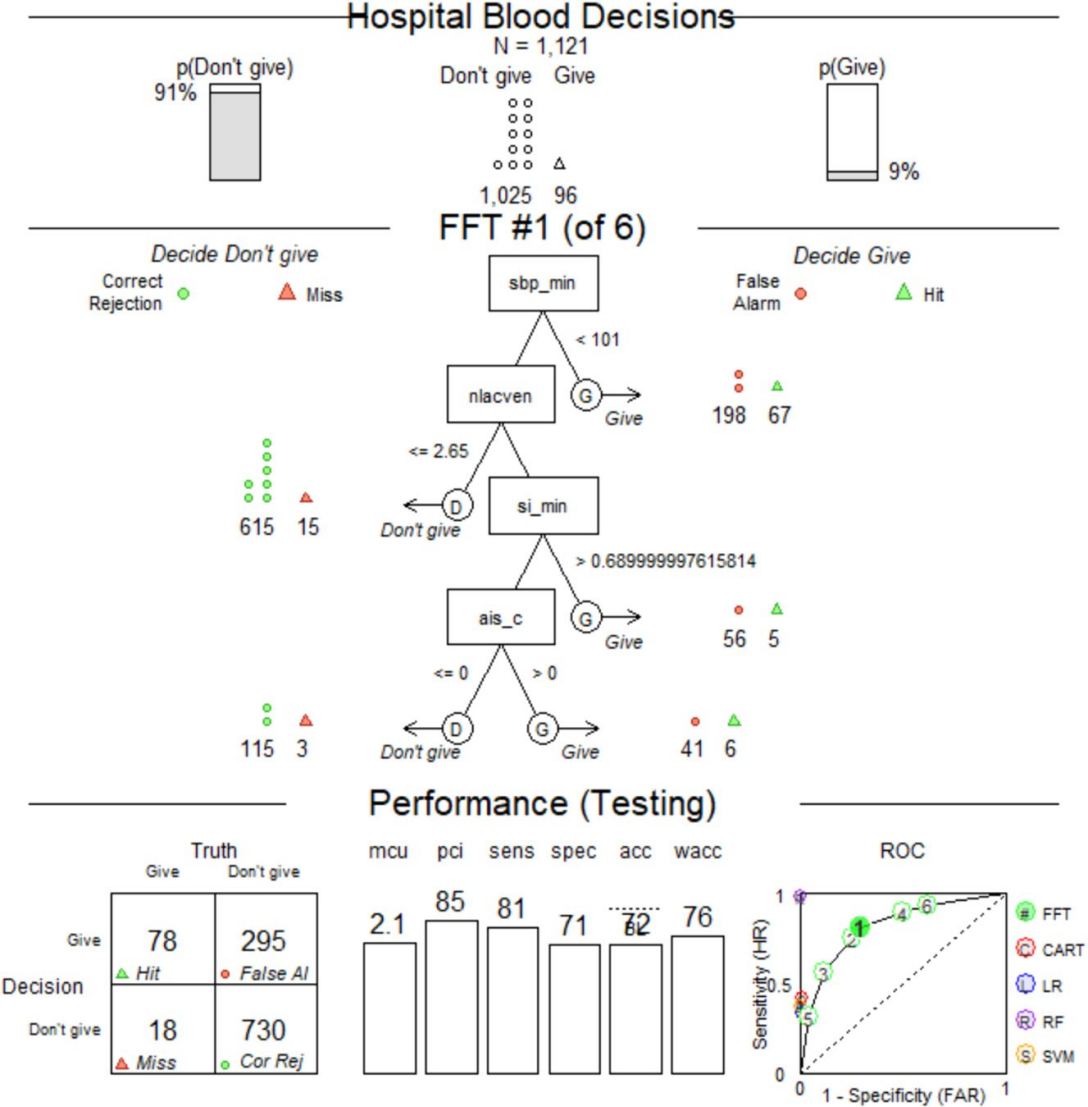
Pilot FFT chosen by the algorithm. The top panels show the number of observations and outcome (4-h hospital blood administration). SBP_min – minimal SBP (mmHg), nlacven – prehospital lactate concentration (mmol/L), si_min – minimum SI (bpm/mmHg), ais_ab – AIS for abdomen (0/1, equal to 1 if the AIS is greater than 2). “Hits” (green triangles) refer to correct blood administrations; “misses” (red triangles) – to incorrect rejections. Sensitivity (triangles) = Hits / (Hits + Misses). Correct rejections (green circles) refer to correct decisions to *not* give blood, false alarms (red circles) – to false positives, or incorrect decisions to give blood. Specificity (circles) = Correct Rejections / (Correct Rejections + False Alarms). A pilot FFT was obtained using training and testing datasets (the testing dataset *N* = 1,121) and selected from a “fan” of possible trees as having the best balance between sensitivity and specificity. A default sensitivity weight of 0.5 resulted in a “zig-zag” shape with alternating decisions. The ROC panel shows a comparison of parameters for the resulting FFT and other common model-building approaches: CART (C, red), Logistic Regression (LR, blue), Random Forest (RF, purple) and Support Vector Machine (SVM, yellow)

The algorithm generated four variables highly associated with hospital blood transfusions within 4 h of arrival (Fig. 2). The variables chosen by the algorithm were evaluated in the following sequence: 1) minimum SBP (continuous), 2) prehospital venous lactate (continuous), 3) minimal SI (continuous), and 4) AIS chest > 2 (categorical). The predictors that were not selected by the *FFT* algorithm were 1) age, 2) mission type (scene or interfacility transfer), 3) AIS head > 2, and 4) AIS lower extremities > 2. The sensitivity for this FFT was 0.81 and specificity 0.71 based on data-driven variable sequence and thresholds.

We applied the FFT definitions from the pilot experiment with rounded thresholds to the entire study population and got similar performance (Supplemental Figure 1A, sensitivity = 0.84, specificity = 0.70). Next, we maximized the sensitivity parameter with an aim to administer hospital blood to the greatest number of eligible patients while minimizing erroneous administrations. Setting the weighting parameter to any value in 0.7–1 range resulted in a “positive-rake” FFT that made positive blood decisions after every node (Supplemental Figure 1B, sensitivity = 0.93, specificity = 0.39). Also, from Supplemental Figure 1B, we can see that the Positive Predictive Value (PPV) for our model is 14.0% (192 / 1,373), while the Negative Predictive Value is 98.1% (769 / 784), confirming that our model rarely mis-identifies a patient needing 4-h hospital transfusion.

The resulting FFT out-performed other model-building approaches (e.g., CART and logistic regression (LR)) by creating a decision support model for early hospital blood administration with higher sensitivity and specificity (Supplemental Figure 1B). Finally, we altered the tree definitions with conventional thresholds used in current field triage guidelines and the literature to simplify for potential use in the prehospital environment [13].

The FFT algorithm found variable thresholds that were different from conventional ones (Fig. 2). We explored thresholds already in common use (i.e., SBP threshold of 90 mmHg and prehospital lactate of 4 mmol/L) or based on ease of calculation for the prehospital provider (SI > 1 = HR > BP) [14]. Applying conventional thresholds (Supplemental Figure 1C) instead data-driven ones (Supplemental Figure 1B) to the dataset greatly reduces the sensitivity but increases the specificity parameter. We tested (a) how altering the FFT definition with conventional thresholds would influence the sensitivity and specificity parameters (Supplemental Figure 1C, Table 2, first blue row) and (b) if a balance between specificity and sensitivity can be reached by using a combination of conventional and newly found thresholds (Table 2, yellow rows). The trees were created the same way as in Supplemental Figure 1B (Table 2, first row) differing only by the threshold values (thresholds and parameters of FFT from Supplemental Figure 1B are highlighted orange in Table 2). Table 2 illustrates how varying the threshold for SBP, lactate, and shock index alters the sensitivity, specificity, and overall performance based on Youden's J index. As expected, using a higher SBP, lower lactate, or lower SI threshold increases sensitivity but decreases specificity.

**Table 2:**
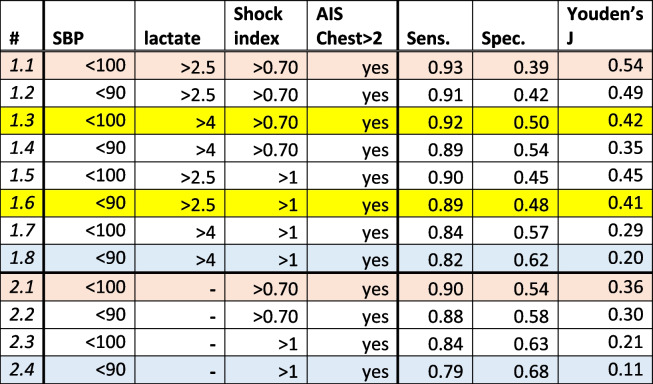
Effect of using deduced, conventional, or mixed thresholds on FFT parameters. **#**- indicates the FFT model number; models with number ‘2.1’ or higher did not include lactate as a variable; Sens. – sensitivity, Spec. – specificity. Youden’s J statistic = sensitivity + specificity – 1 summarizes the performance of each model

Tree from row 1.1 is depicted in Supplemental Figure 1B

Tree from row 1.8 is depicted in Supplemental Figure 1C

We performed sensitivity analyses by removing the lactate term from the models and using FFT-derived vs. conventional thresholds for SBP and SI (Table 2, rows 2.1–2.4), recognizing that prehospital lactate may not be widely available. The sensitivity was often higher for the models containing the lactate term (compare rows 2.1 and 1.1/3, 2.2 and 1.2/4, 2.3 and 1.5/7, 2.4 and 1.6/8), but the specificity and Youden’s J index were lower.

We also assessed current practice of prehospital blood transfusion by the critical care service and the need for early in-hospital transfusion. Table 3 shows a cross-tabulation of actual prehospital blood administration by early hospital transfusions. Of 207 subjects who required early hospital transfusions, 79 (38.2%) subjects also received blood before arriving to the hospital (Table 3, upper left quadrant). The majority (73) of these 79 subjects had SBP < 90 mmHg and received prehospital blood according to the prehospital care service protocol for blood transfusions. Among 60 patients who received prehospital transfusions but did not require hospital blood (Table 3, upper right quadrant), 33 (55.0%) patients had SBP < 90 mmHg. Patients who received blood with systolic blood pressures > 90 mmHg, either received the product on the order of the physician or in deviation from the protocol.

**Table 3:** Cross-tabulation of prehospital transfusions by 24-h hospital transfusions

	4 h hospital ED blood = YES	4 h hospital ED blood = NO
Prehospital blood = YES	79 (3.66%)	60 (2.78%)
Prehospital blood = NO	128 (5.93%)	1,890 (87.6%)

In our Bayesian analysis, the most predictive model demonstrated statistically significant associations with tachycardia (OR = 1.74; 95% CI 1.12 – 2.55), elevated prehospital lactate (OR = 2.31; 95% CI 1.55 – 3.37), and hypotension (OR = 11.59; 95% CI 7.70–16.98) for early in-hospital transfusion. In the Bayesian subgroup analysis of patients with SBP > 90 mmHg (N = 1,901; 87.6%), the most predictive model included minimum shock index (OR = 25.6; 95% CI 2.54 – 113.2), elevated lactate (OR = 2.17; 95% CI 1.11 – 3.77), and tachycardia (OR = 1.59; 95% CI 0.72 – 2.94). Based on the 95% credible intervals, in the hypotensive cohort lactate and minimum shock index were significantly associated with a higher posterior probability of early in-hospital transfusion.

**Fig. 3:**
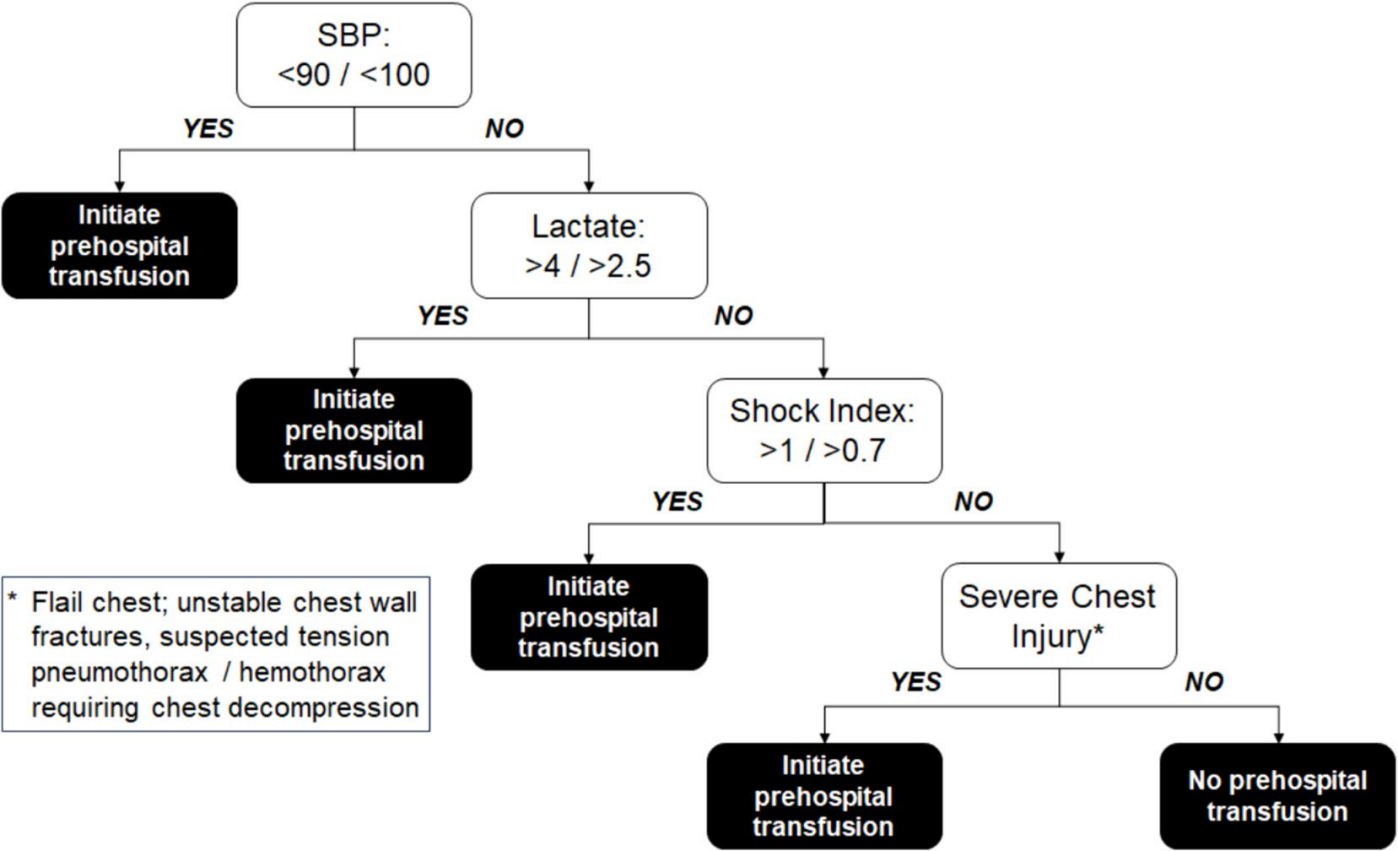
Summary of obtained decision rules and how they may guide prehospital transfusions. The rules were obtained based on the need for 4-hour in-hospital transfusions

Synthesizing and operationalizing the results from our approaches for potential field use, we developed an algorithm for prehospital blood transfusion that incorporates prehospital SBP, prehospital lactate, shock index, and severe abdominal injuries (Fig. 3). This algorithm allows for different threshold values that may be tailored according to system resources and time considerations.

We also applied the FFT definitions from Supplemental Figure 1B but excluding the node for severe chest injuries (Supplemental Figure 2). The resulting sensitivity and specificity parameters were slightly lower than those of the four-factor model (Supplemental Figure 1B, Supplemental Figure 2).

## Discussion

Using advanced statistical methods to control for confounders and to maximize the information provided by a large cohort of adult trauma patients with granular prehospital data, we identified four variables that predict early in-hospital transfusions. These variables, which are accessible to prehospital clinicians, were selected by an FFT algorithm to facilitate the decision to administer prehospital blood quickly with a parsimonious (small) set of data. We confirmed these findings using Bayesian analysis to identify strong predictors of early in-hospital transfusion. Prehospital lactate emerged as a strong predictor for transfusion need from both the FFT and Bayesian approaches among patients who were not hypotensive. This is consistent with recent study by *Griggs *et al*.* who also predicted in hospital transfusion using prehospital lactate concentration [15].

Administration of prehospital blood products to patients in hemorrhagic shock reduces mortality [4]. A systematic review and meta-analysis by Rijnhout et al. describes the administration of prehospital blood products as feasible and safe, but describes the evidence as low-quality and difficult to compare because there is no standard indication for transfusion [16]. While tools have been developed to identify patient at risk of Trauma Associated Severe Hemorrhage (TASH) and for massive transfusion (ABC score), they rely on data not readily available in the prehospital environment (hemoglobin and ultrasound) and neither was developed for the prehospital environment [17, 18].

To find the simplest decision model to identify people who need prehospital blood transfusions, we are faced with two competing considerations: 1) correctly identifying the greatest number of people who need blood (i.e., maximizing the sensitivity of the model), and 2) conserving limited resources of blood. Using these considerations, an EMS Medical Director may conclude that the model with a lactate concentration threshold of 2.5 mmol/L (Sensitivity = 0.89, Specificity = 0.48, Table 2 row 1.6) is more appropriate for use in a rural setting with delayed access to a trauma center and subsequent damage control resuscitation, while a model with a 4 mmol/L threshold (Sensitivity = 0.82, Specificity = 0.62, Table 2 row 1.8) could be more suitable for urban settings with short prehospital times. Similar trade-offs can be made with the thresholds for SBP and shock index.

We adjusted the model thresholds to create simple rules for quick reference in the field (Table 2). The results depicted in Table 2 have broad implications for prehospital clinicians, ranging from urban and rural EMS systems to austere military environments that might require prolonged field care. Using the four variables derived from our models, prehospital system leadership can decide what thresholds are appropriate for transfusions in their respective systems, based on existing resources and trauma center access.

Previous studies associate prehospital lactate with mortality and morbidity in trauma patients [19, 20]. Subsequent work demonstrated the association between lactate and need for life saving interventions [21]. Recent work by Fukuma et al. and Galvagno et al. established that prehospital lactate threshold of > 4 mmol/L is associated with the need for life-saving interventions for hemorrhage control [14, 22]. This threshold is more conservative than the one found by the FFT algorithm (2.5 mmol/L).

The last cue identified to trigger potential transfusion is severe chest injury. In the data we used AIS > 2; however, recognizing this is not available in the field setting as an objective number, this cue would rely on clinical exam evidence, much like the anatomic triage criteria for the national field triage guidelines are identified. We suggest operationalizing this cue as flail chest, unstable chest fractures, or need for needle decompression (Fig. 3). Local medical directors certainly would have discretion to operationalize this cue in an alternative way given the personnel, resources, and trauma population seen by his or her EMS agency. We do show comparable accuracy if the cue is omitted (Supplementary Figure 2), allowing further adaptation to local circumstances given it is the most subjective cue in operational form.

A key limitation of our study is that decision to transfuse blood is not always synonymous with the need to transfuse blood. Also, our analyses are retrospective and derived from a single EMS agency serving a regional trauma system. The dataset was limited to patients who had lactate sampled, which imparts bias among patients with hemorrhagic shock. Selection bias may result when treatment priorities preclude sampling of lactate in the sickest patients. EMS data is rarely entered into the record contemporaneously with care and is subject to recall and reporting bias. We import data electronically (vital signs, times and point of care testing) into the prehospital health record which mitigates these biases. There is likely a selection and sensitivity bias, as our critical care organization is called for patients with more severe injuries or those who are geographically distant from trauma care.

## Conclusion

We developed a parsimonious, clinically relevant algorithm to identify patients who may require prehospital transfusion. This algorithm accounts for prehospital lactate concentration, which is useful for identifying patients with occult shock not meeting the conventional threshold for hypotension. Thresholds of decision factors should be adjusted to meet the needs and resources of a given prehospital trauma system. Further work is necessary to externally validate this algorithm for prehospital blood transfusion.

We are including the Appendix describing the FFT algorithm, Blood Administration protocol, and the study checklist for adhering to STROBE guidelines as Supplemental Digital Content.

### Supplementary Information

Supplementary Material 1: Supplemental Figure 1. Applying FFT definitions to the whole population The following FFTs were obtained by applying pilot (A, B) or conventional (C) thresholds to the entire dataset (*N*=2,157). Pilot thresholds were chosen by the algorithm (see Fig. 2). A. FFT performance with simplified (rounded) thresholds. B. A refined FFT obtained by increasing the sensitivity weight parameter. A sensitivity weight between 0.7 and 1 resulted in a “positive rake” shape with positive decisions after each node (rectangle). The inset shows a comparison of ROC parameters for the resulting FFT and other common model-building approaches: CART (C, red), Logistic Regression (LR, blue), Random Forest (RF, purple) and Support Vector Machine (SVM, yellow). The latter two approaches are aggregate methods that cannot be used for comparison here because only one tree is built using tree definitions from the data-driven pilot experiment from Fig. 2. C. FFT performance with conventional thresholds.

Supplementary Material 2: Supplemental Figure 2. FFT performance without the chest AIS cue.

Supplementary Material 3.

## Data Availability

Data is available upon request.

## Abbreviations

AIS: Abbreviated Injury Scale
bpm: Beats per minute
ED: Emergency Department
EHR: Electronic Health Record
EMS: Emergency Medical Services
ISS: Injury Severity Score
SBP: Systolic Blood Pressure
SI: Shock Index
TC: Trauma Center
ROC: Receiver Operating Characteristics
FFT: Fast Frugal Tree
