# Supplementary material for: Identifying trigger cues for hospital blood transfusions based on ensemble of machine learning methods
Source: Int J Emerg Med. 2024 Jun 19;17:76. doi: 10.1186/s12245-024-00650-0 (PMC11186116; doi:10.1186/s12245-024-00650-0)
Supplement: Supplementary file 1 — Supplementary Material 1: Supplemental Figure 1. Applying FFT definitions to the whole population The following FFTs were obtained by applying pilot (A, B) or conventional (C) thresholds to the entire dataset (N=2,157). Pilot thresholds were chosen by the algorithm (see Fig. 2). A. FFT performance with simplified (rounded) thresholds. B. A refined FFT obtained by increasing the sensitivity weight parameter. A sensitivity weight between 0.7 and 1 resulted in a “positive rake” shape with positive decisions after each node (rectangle). The inset shows a comparison of ROC parameters for the resulting FFT and other common model-building approaches: CART (C, red), Logistic Regression (LR, blue), Random Forest (RF, purple) and Support Vector Machine (SVM, yellow). The latter two approaches are aggregate methods that cannot be used for comparison here because only one tree is built using tree definitions from the data-driven pilot experiment from Fig. 2. C. FFT performance with conventional thresholds. [file 12245_2024_650_MOESM1_ESM.docx]

**Appendix/Supplemental Methods**

The FFT algorithm (*ifan*) was created in 2017 in R language by Nathan Phillips.^11^ The algorithm works by first evaluating individually each predictor variable/cue on how well it partitions the outcome variable. As part of this evaluation, the algorithm applies various thresholds to the cue and finds the one that best separates the outcome. Afterwards, the algorithm choses a small number (default = 4) of cues (and their thresholds) that are the most powerful separators, and uses this information to create a “fan” of decisions trees, where the number of trees is 2^(number of best-separating cues – 1). The procedure abstracts two parameters from each tree: Sensitivity (how well the tree identifies positive cases, here, subjects who need hospital blood) = Correct Acceptances (i.e., “hits”) / (Correct Acceptances – Incorrect Rejections (i.e., false negatives or “misses”)), and Specificity (how well the tree identifies the negative cases, here, subjects who do *not* need hospital blood) = Correct Rejections / (Correct Rejections – Incorrect Acceptances (i.e., false positives or “false alarms”)).

As end result, the algorithm selects an optimal tree from this fan of trees based on the weighted accuracy criterion (wacc) = Sensitivity*weight + Specificity*(1 – weight). By default, the weight (sens.w parameter) is equal to 0.5 for a balance between Sensitivity and Specificity. This weight creates a “zig-zag” FFT that alternates positive and negative decisions after evaluating each variable/cue. To maximize the Sensitivity parameter, we increased the sensitivity weight (sens.w) above 0.5 (0.7-0.9) resulting in a “positive-rake” FFT that makes a positive decision after each node.
