# Supplementary figures and images for: Identifying trigger cues for hospital blood transfusions based on ensemble of machine learning methods
Source: Int J Emerg Med. 2024 Jun 19;17:76. doi: 10.1186/s12245-024-00650-0 (PMC11186116; doi:10.1186/s12245-024-00650-0)

**
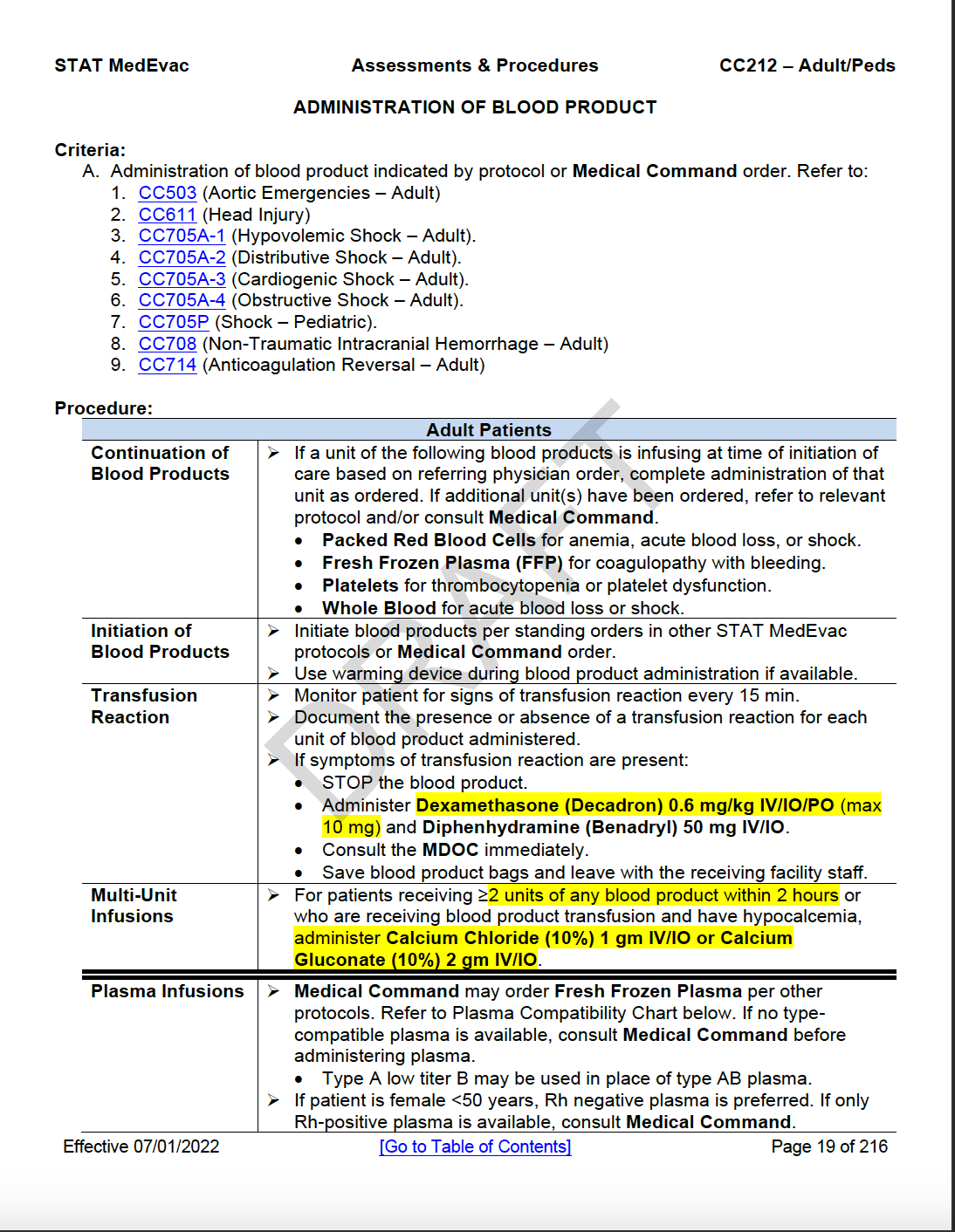
**

**
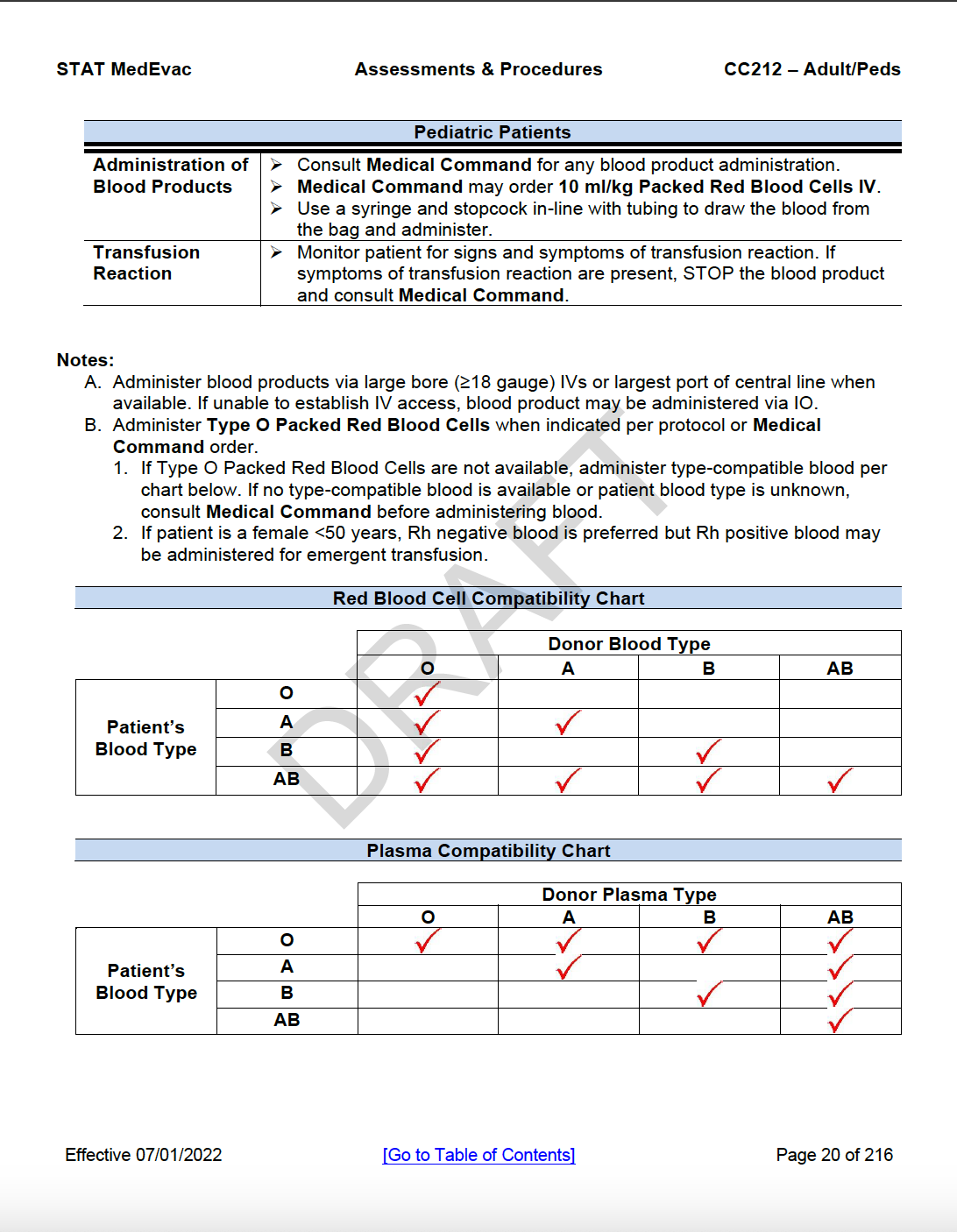
**

**
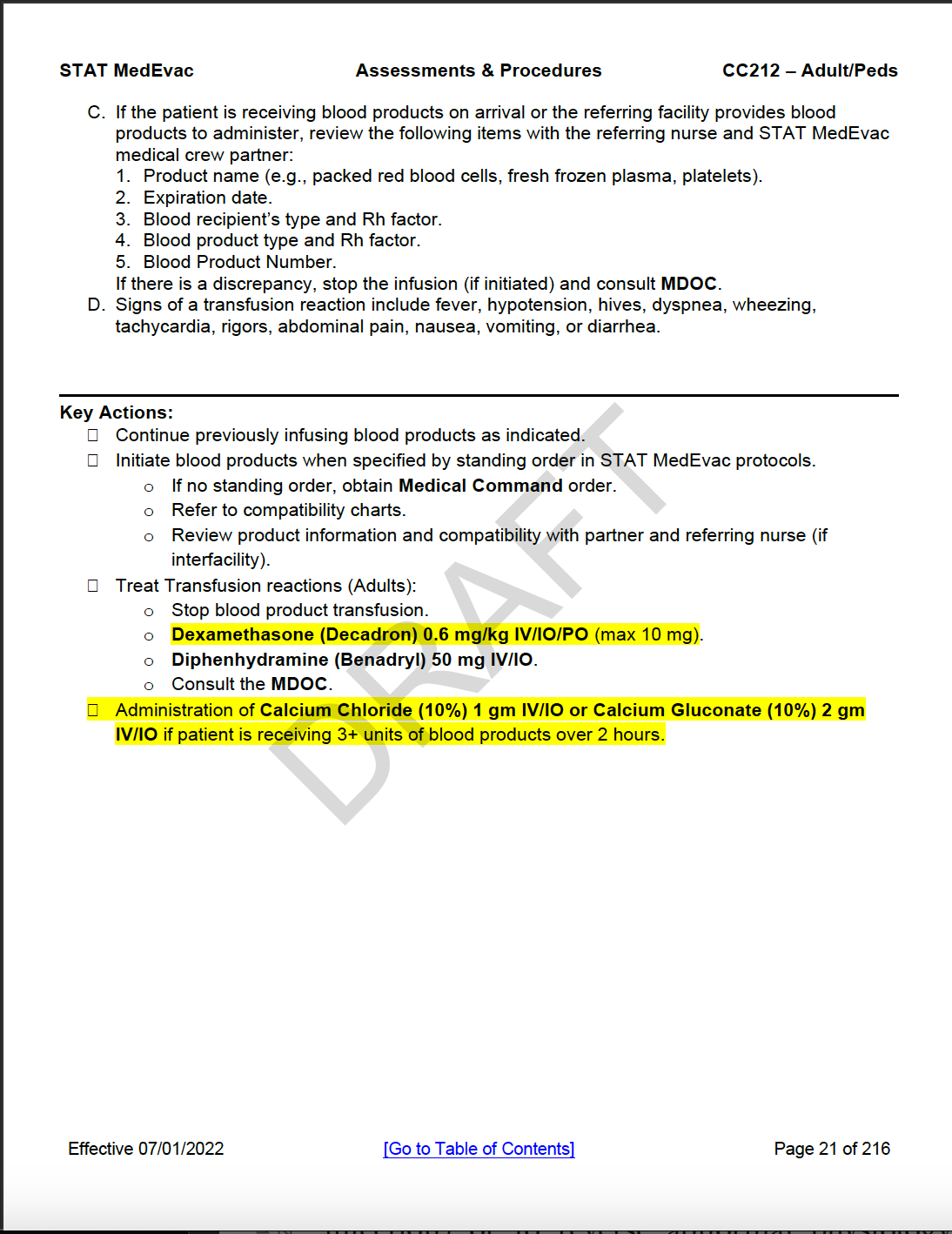
**

Supplement: Supplementary file 2 — Supplementary Material 2: Supplemental Figure 2. FFT performance without the chest AIS cue. [file 12245_2024_650_MOESM2_ESM.docx]
