# Supplementary material for: Identifying trigger cues for hospital blood transfusions based on ensemble of machine learning methods
Source: Int J Emerg Med. 2024 Jun 19;17:76. doi: 10.1186/s12245-024-00650-0 (PMC11186116; doi:10.1186/s12245-024-00650-0)
Supplement: Supplementary file 3 — Supplementary Material 3. [file 12245_2024_650_MOESM3_ESM.docx]

| **Supplemental Table 1. Protocol for HEMS RBC transfusion** |
| --- |
| RBC transfusion should be administered if any one of the following are present: |
| hypotension with systolic blood pressure <90mmHg and evidence of hemorrhagic shock as suggested by the following:   1. Changes in mental status 2. Changes in skin color (pallor, mottling or cyanosis) 3. Tachycardia with heart rate >120 beats per minute 4. Capillary refill >2 seconds 5. Urine output <30 ml/hour for ≥4 hours (inter-facility transports) 6. Lactate level ≥4 mmol/L 7. Shock index (HR/SBP) >0.9 8. RBC transfusion initiated at a referring facility (inter-facility transports) |
|  |
| In cases of penetrating wounds or clinical evidence of active bleeding, RBC may be initiated earlier through consultation with a medical command physician. |
| HEMS, Helicopter emergency medical services; RBC, Red blood cells; HR, Heart rate; SBP, Systolic blood pressure |
